# The Effect of Multi-Walled Carbon Nanotubes on the Material Properties of Polyamide 66 Nanocomposites

**DOI:** 10.3390/polym17101319

**Published:** 2025-05-12

**Authors:** Ionut-Laurentiu Sandu, Felicia Stan, Catalin Fetecau, Adriana-Madalina Turcanu, Alina Cantaragiu Ceoromila, Andrei-Mihai Prada, Florin-Sandu Blaga

**Affiliations:** 1 Center of Excellence Polymer Processing, Dunarea de Jos University of Galati, 47 Domneasca, 800 008 Galati, Romania; laurentiu.sandu@ugal.ro (I.-L.S.); felicia.stan@ugal.ro (F.S.); madalina.constantinescu@ugal.ro (A.-M.T.); 2 Cross-Border Faculty, Dunarea de Jos University of Galati, 47 Domneasca, 800 008 Galati, Romania; alina.cantaragiu@ugal.ro; 3 Industrial Engineering Department, Faculty of Managerial and Technological Engineering, University of Oradea, 1 Universitatii, 410 087 Oradea, Romania; andrei.prada@plastor.ro (A.-M.P.); fblaga@uoradea.ro (F.-S.B.)

**Keywords:** polyamide 66, multi-walled carbon nanotubes, nanocomposites, injection molding, characterization, thermo-mechanical behavior, rheological behavior, annealing

## Abstract

The aim of this study was to evaluate the effect of multi-walled carbon nanotubes (CNTs) on various material properties of the polyamide 66 (PA66) nanocomposites. This is achieved first by investigating the effect of CNTs (0.1–5 wt.%) on the material properties of PA66 pellets, and second, on the injection-molded PA66/CNT nanocomposites. Thermal analysis revealed that CNTs do not have a significant effect on the melting behavior and melting temperature of PA66/CNT nanocomposites, but they increase the crystallization temperature of the nanocomposites. Rheological analysis showed that the melt shear viscosity of the PA66 increased with increasing CNT content particularly above 1 wt.%. Additionally, the PA66 nanocomposites exhibit shear-thinning behavior, and this effect is more significant at higher CNT contents. The FT-IR analysis revealed the absence of chemical bonds between PA66 and CNTs and, consequently, the uniform dispersion of CNTs in the PA66 matrix. Mechanical testing indicated that the inclusion of CNTs (0.1 to 5 wt.%) in PA66 matrix could not improve the tensile modulus to a great extent, while it decreased the ultimate tensile strength of PA66 nanocomposites under tension. On the other hand, CNTs positively influenced the mechanical behavior under bending (+15% increase at 5 wt.%). Among the nanocomposites, PA66 filled with 5 wt.% CNTs exhibited the optimal mechanical performance in terms of tensile strength (58 MPa), tensile modulus (2689 MPa), bending modulus (2072 MPa), and bending strength (104 MPa). The experimental results also showcase the significant improvement in the tensile and bending mechanical properties of the injection-molded PA66 nanocomposites after thermal annealing at −40 °C and 180 °C for one hour. This experimental study provides guidelines for the structure–property–processability of the PA66 nanocomposites, revealing the complex relationship between the CNTs and the enhancement of mechanical properties, while highlighting the potential of thermal annealing in improving the mechanical performance of PA66 nanocomposites. This will be further investigated to promote the use of PA66 nanocomposite in industrial applications.

## 1. Introduction

Carbon nanotube (CNT)-reinforced polymer nanocomposites are nowadays used in various end-user industrial applications, including automotive parts and sporting goods, as an alternative to traditional short carbon fiber (CF)-reinforced polymer composites [1,2], given the distinctive structure (i.e., single, double, and multiple walled tubes with aspect ratio in the range of several thousand), excellent mechanical properties (Young’s modulus up to 1 TPa, and high tensile strength up to 60 GPa), electrical resistivity like copper (about 10^−8^ Ω·m), very low density (1.3–2 g/cm^3^), and high thermal conductivity (up to 6000 W/mK) [3,4,5] of CNTs.

Experimental studies on polymer/CNT nanocomposites have shown that the addition of a small amount of CNTs into a thermoplastic polymer (e.g., up to 5 wt.%) generally improves the mechanical properties of the nanocomposite, such as elastic modulus, tensile strength, or stress at break [5,6,7,8,9,10,11,12,13,14,15]. Moreover, CNTs also enhance the thermal [14,15,16,17,18] and electrical [9,10,11,12,13,14,19] conductivities of the polymer/CNT nanocomposites as compared with the neat polymers. For example, with the addition of up to 5 wt.%, the conductivity of the polymer/CNT nanocomposites can reach at least the semiconducting domain [12,13,14].

However, CNTs have a natural tendency to form clusters due to very high van der Waals forces and do not always have a positive effect on the polymer/CNT nanocomposites. Studies on the rheological behavior of polymer/CNT nanocomposites have shown that the melt shear viscosity increases with increasing CNT loading [5,12,14,15,20,21], which is detrimental for the manufacturing processes such as injection molding, extrusion, or 3D printing. On the other hand, the mechanical properties of polymer/CNT nanocomposites largely depend on the interfacial adhesion, dispersion, and distribution of the nanotubes in the polymer matrix, as well as the degree of crystallinity [4,5,22,23,24,25,26,27,28,29,30,31,32,33,34], all of which are interdependent. It was reported that weak CNT–polymer interfacial bonding, poor CNT dispersion, and agglomeration can decrease the mechanical properties (e.g., Young’s modulus or tensile strength [24,25,26,27] and ductility [13,14,35,36,37]) and increase the electrical percolation threshold [20,28,38,39,40]. Additionally, CNT agglomerates could restrict the formation of heterogeneous nuclei, decreasing crystallinity and, hence, the mechanical properties [41].

Therefore, the main challenges with CNTs are to ensure uniform dispersion and distribution within the polymer matrix during processing. To mitigate these challenges and improve interfacial adhesion, as well as prevent the formation of CNT agglomerates, several strategies were adopted such as functionalization [4], ultrasonication and dispersion methods, employing surfactants [29] and synthesis methods (arc discharge, chemical vapor deposition (CVD), and laser ablation) [5].

Polyamide 66 (PA66) is one of the most commonly used polyamides in industrial applications [42,43,44,45] that require high performance, such as high strength and toughness, good impact strength, thermal stability, and excellent chemical resistance, among other properties [46,47,48,49]. If the end-user applications (e.g., aerospace and automotive parts and components, armor, protective clothing, bullet-proof panels, and sports equipment [50,51,52]) require enhanced strength and stiffness as well as long-term stability under constant or fatigue loading, different types of carbon fibers (i.e., long, short, or unsized) are added into the PA matrix [19,53,54,55,56,57,58,59,60,61]. The mechanical properties of the PA66/CF composites may be further enhanced by improving the quality of the interfacial bonding between the fibers and the polymer matrix through surface treatment of the CFs (i.e., oxidation, plasma treatment, etc.) [23]. However, the main disadvantage of the PA66/CF composites is the high CF content needed to improve the mechanical properties. To increase the ultimate tensile strength or elastic modulus of the PA66 matrix, CFs in mass fractions of up to 50–60 wt.% were reported [54,55], which increases the weight of products and components.

Although CNTs have the ability to simultaneously reduce the overall weight and significantly improve the electrical, thermal, and mechanical properties of the polymers at lower CNT loading compared to CFs [19,53], studies on the effect of CNTs on the material properties of PA66 nanocomposites including the effect of manufacturing methods are scarce [8,62,63,64,65,66,67,68,69,70].

The literature review indicates that most of the studies on PA66/CNT nanocomposites, so far, have investigated the possibility of preparing PA66/CNT nanocomposites with different CNT contents and investigated the effect of CNTs on the thermal [62,63,64,65,66,67,68,69] rheological [64,65,68], electrical [64], and mechanical properties [8,62,63,66,67,70].

Thermal analysis indicated that the incorporation of CNTs did not affect the melting behavior of PA66 and PA66 nanocomposites [62,63,64], but promoted the crystallization and increased the crystallization temperature of the PA66 nanocomposites as compared with the neat PA66 [63,66]. On the other hand, it was reported that the crystallinity of PA66/CNT nanocomposites could increase slightly [63,66,67,68,69] or show no clear dependency [62] with the addition of CNTs. Mechanical testing revealed that addition of a small amount of CNTs (0.5 to 1 wt.%) could enhance the elastic modulus and ultimate tensile strength of the PA66 nanocomposites by about 32% and 43%, respectively, for 0.5 wt.% CNTs [62], and by about 24% and 12%, respectively, for 1 wt.% [63]). CNTs also improve the electrical properties of PA66 nanocomposites [64]. The addition of 2–5 wt.% of CNTs increased the electrical volume resistivity of the nanocomposites by about 12–14 orders of magnitude as compared to the neat PA66 [64]). On the other hand, it was reported that the addition of 3 wt.% CNTs increased the storage modulus and the complex viscosity compared to the neat PA66 [65]).

Knowledge of structure–property–processability of PA66 nanocomposites may unlock new industrial applications, particularly injection-molded lightweight components that require high strength at elevated temperatures. Therefore, in this study, a systematic investigation is carried out using different characterization methods to assess the effects of CNTs on different material (thermal, physical, rheological, morphological, and mechanical) properties of PA66/CNT nanocomposites with 0.1–5 wt.% CNTs. Given the practical importance, the effect of short-term annealing on the thermal and mechanical properties of injection-molded specimens was also assessed.

## 2. Experimental

### 2.1. Raw Materials

The nanocomposites investigated in this study are based on a polyamide 66 (Altech PA66 A 1000/109, Albis Plastics GmbH, Hamburg, Germany [71]) and thin multi-walled carbon nanotubes (NC7000, Nanocyl S.A., Sambreville, Belgium) [72]. The PA66 nanocomposites with 0.1, 0.3, 0.5, 1, 3, and 5 weight percent (wt.%) of CNTs were supplied by Nanocyl S.A. (Sambreville, Belgium). PA66 nanocomposites were obtained from PLASTICYL™ PA1501 masterbatch with pre-dispersed 15 wt.% CNTs [73]. According to the manufacturer, the nanocomposite compounds were processed by diluting the masterbatch using a 48 L/D ratio twin-screw extruder under proprietary conditions [73].

The PA66 matrix is a semi-crystalline thermoplastic polymer with water and humidity absorption rates of 8.5 and 2.8%, respectively, a density of 1130 kg/m^3^, and a melting temperature of 252 °C (dry) [71,74]. The NC7000 are produced via the catalytic carbon vapor decomposition (CCVD) with the following main properties [72,75]: average nanotube diameter of 9.5 nm, average nanotube length of 1.5 μm, carbon purity > 90%, surface area 250–300 m^2^/g, volume resistivity 10^−4^ Ω cm, metal oxide 10%, tensile strength 10–60 GPa, and Young’s modulus 1 TPa.

### 2.2. Fourier Transformation Infrared Spectrometry

Fourier transformation infrared (FT-IR) spectrometric measurements were performed using a Nicolet™ iS50 FT-IR spectrometer (Thermo Fisher Scientific Inc., Waltham, MA, USA) equipped with a built-in ATR accessory, DTGS detector, and KBr beam splitter. A number of 32 scans were co-added over the range of 4000–400 cm^−1^ with a resolution of 4 cm^−1^.

### 2.3. Differential Scanning Calorimetry

The thermal behavior of PA66 and PA66 nanocomposites was analyzed using a thermal analysis system (DSC 3 STAR^e^ System, Mettler Toledo, Columbus, OH, USA). Samples (of about 8–12 mg) from PA66 and PA66/CNTs pellets and injection-molded specimens were sealed into a 40 µL Al crucible with a pin and subjected to multiple heating and cooling scans at a heating/cooling rate of 10 °C/min in a temperature range of 20–320 °C. The first cooling and the second heating scans were used to extract the thermal properties. The glass transition temperature, *T_g_*, was extracted using the standard DSC analysis routine (i.e., midpoint option on the first cooling). The crystallization temperature, *T_c_*, and melting temperature, *T_m_*, were extracted as the peak of the first exothermic and second endothermic curves, respectively. The melting enthalpy, Δ*H_m_*, computed as the integral of the second endothermic area, was used to calculate the degree of crystallinity [13,14]

(1) $$\chi_{c}=\frac{1}{1-\mathrm{wt}.\%}\cdot \frac{\Delta H_{m}}{\Delta H_{m}^{0}}\times 100\;(\%),$$

where $\Delta H_{m}^{0}$ is the melting enthalpy of 100% crystalline PA66 (196 J/g [76]) and ϕ is the CNT weight fraction (wt.%).

### 2.4. Bulk Density

The solid-state density of the PA66 and PA66/CNT nanocomposites was determined based on Archimedes’ principle using an analytical balance (AB204-S/FACT, Mettler Toledo, Columbus, OH, USA) equipped with a kit density, following ISO 1183-1 standard [77]. The PA66 and PA66/CNTs pellets and injection-molded samples were weighed in ethanol and air. To determine the measurement variability, ten independent determinations were performed.

### 2.5. Rheological Characterization

#### 2.5.1. Melt Flow Testing

To assess the flow behavior of the PA66 and PA66/CNTs nanocomposites, melt flow index (MFI) measurements were performed based on the ISO 1133 test method [78] using an extrusion plastometer (Melt Flow Quick Index, Instron Corp., Norwood, MA, USA). The extrusion temperature was set to 275 and 280 °C under a load of 1.2 kg. Since the nanocomposite with 5 wt.% CNTs have very high viscosity, the MFI was measured at a load of 3.8 kg. Moisture has a negative effect on the melt processing of polymers and polymer/CNT nanocomposites, leading to defects [79,80,81,82]. In general, for PA66, it is recommended that the moisture content be lower than 0.08% [74]; therefore, before MFI testing, the PA66 and PA66/CNTs pellets were dried at 100 °C for 48 h using a vacuum oven (EV-50, Raypa, Barcelona, Spain).

#### 2.5.2. Capillary Rheometry

The effect of the CNT wt.% on the melt shear viscosity of PA66 was examined using a capillary rheometer (RG75, Göttfert, Buchen, Germany) equipped with a capillary die with a length-to-diameter ratio (L/D) of 30:1. The apparent melt shear viscosity was measured at 275 °C and shear rates from 100 to 5000 1/s, in a random order. For all rheological tests, the melting time was set to 10 min. It should be noted that the pellets were dried under the same conditions as in the case of MFI.

### 2.6. Injection Molding

**Figure 1:**
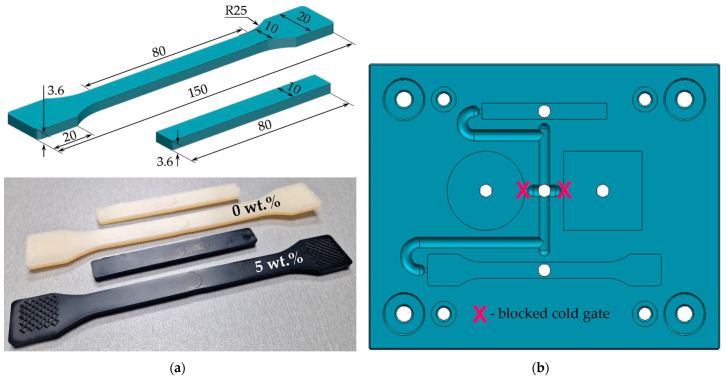
Geometry and dimensions (in mm) of the (**a**) dog-bone (tensile) and three-point bending specimens; (**b**) injection-molding cavity plate.

The PA66 and the PA66/CNT nanocomposites were injection-molded into dog-bone and three-point bending specimens according to ISO 527 (type 1B) [83] and ASTM D790 [84] standards, respectively. Figure 1 shows the geometry and dimensions of the samples as well as the injection-molding cavity plate.

The injection molding process was performed on an electric injection molding machine (Allrounder 370 E Golden Electric, Arburg, Lossburg, Germany) with a screw diameter of 25 mm and a clamping force of 600 kN. The PA66 and PA66/CNT pellets were conditioned for 4–6 h at 100–120 °C using an industrial granulate dryer (T50 IM482, Piovan, Italy, Santa Maria di Sala, Venice, Italy) until the humidity was less than 0.1%.

Regardless of the CNT content, the injection-molding parameters were set constant as follows: barrel temperature 280 °C, injection-molding temperature (at nozzle) 275 °C, mold temperature 80 °C (i.e., above the glass transition temperature to ensure proper crystallization), injection volume 25 cm^3^, injection speed 30 mm/s, injection pressure 900 bars, holding pressure 400 bars, holding time 8 s, and cooling time 20 s.

### 2.7. Morphological Characterization

The dispersion of the CNTs into the PA66 matrix was investigated using scanning electron microscopy (SEM) (Quanta 200, FEI, Hillsboro, OR, USA) at 15 kV voltage. Injection-molded samples were cryo-fractured in liquid nitrogen and analyzed after gold-based alloy sputtering (e.g., layer of 5–10 nm). Additionally, the fractured surface of the injection-molded samples after the tensile testing was studied after sputtering.

### 2.8. Mechanical Characterization

Tensile and three-point bending tests were performed using a universal testing machine (M350-5AT, Testometric, Rochdale, UK). Tensile tests were carried out at different crosshead speeds (e.g., 1, 10, and 100 mm/min) with an initial distance between the grips of 115 mm, while the three-point bending tests were performed at a crosshead speed of 1 mm/min with a support span of 40 mm. At least five independent samples were tested to calculate the mean and the standard deviation of the mechanical properties according to ISO 527 [83] and ASTM D790 [84] standard test methods, respectively.

### 2.9. Thermal Annealing

PA66 and PA66/CNT nanocomposite samples were annealed in a climatic chamber (VC^3^ 7018, Vötsch, Balingen-Frommern, Germany). The samples were placed on the metal grid and subjected to −40 °C and 180 °C for one hour, at a cooling and heating rate of 2 °C/min. For a semicrystalline polymer, the annealing temperature should be higher than the glass-transition temperature and lower than the melting temperature or close to the in-service maximum temperature. In this study, the temperature of 180 °C was selected as the median between the glass transitions and the end-set of the melting temperature [85,86]. The temperature of −40 °C was selected as the maximum negative temperature at which the PA66 and PA66/CNT nanocomposites could be exposed to operational conditions. The annealed samples were analyzed in terms of DSC thermal and mechanical properties and compared with the as-molded (unannealed) samples.

### 2.10. Statistical Analysis

The main effect plots and analysis of variance (ANOVA) with Tukey pairwise comparison test were used to analyze the experimental data and to assess the effect of each factor at a significance level of 0.05 (e.g., *p*-value < 0.05). The statistical analysis was performed with Minitab software (version 18, Minitab LLC, State College, PA, USA).

## 3. Results and Discussion

### 3.1. FT-IR Spectra

**Figure 2:**
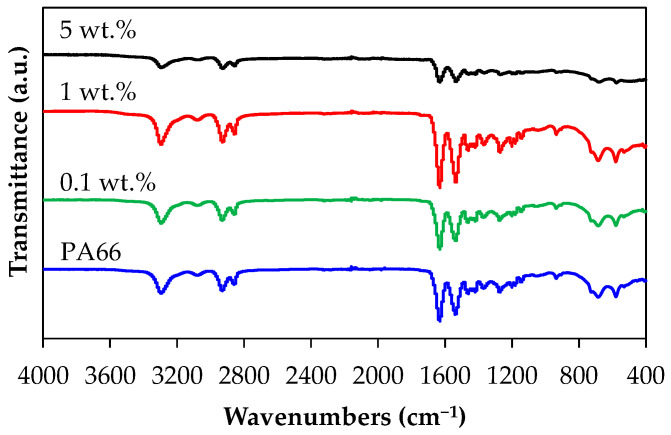
FT-IR spectra of PA66 and PA66 nanocomposites with 0.1, 1, and 5 wt.% CNTs.

The dispersion of CNTs into the PA66 matrix was investigated using FT-IR analysis. The FT-IR spectra of the neat PA66 and PA66/CNT nanocomposites (0.1, 1 and 5 wt.%) are presented in Figure 2. The characteristic polyamide N-H stretch at 3295 cm^−1^ and N-H deformation at 1600 cm^−1^ were observed in the PA66 and PA66 nanocomposite spectra. The sharp bands at 2938–2928 cm^−1^ were attributed to the asymmetric and symmetric C-H stretching variations. The fingerprint region of the spectrum presented dominant sharp peaks at 1630–1640 cm^−1^ due to the C=O stretch of the amide I group (NH-C=O) in PA66 and at 1534–1536 cm^−1^ due to in-plane NH deformation vibration of the amide II. Other bands at 1370 cm^−1^ were specific to the CN stretch and in-plane NH deformation of the amide III; at 1200 cm^−1^, the band specific to the amide III coupled with hydrocarbon skeleton; at 934 cm^−1^, the band was specific to C–C=O stretch; at 720 cm^−1^, the presence of a shoulder was due to hydrogen-bonded secondary amines; and at 684 cm^−1^, the bend was due to N-H bending vibration of the N-C=O group, as previously reported by other researchers [87,88,89,90]. As can be seen in Figure 2, there are no significant variations in both the peak positions and relative intensities for the neat PA66 and PA66 nanocomposites, which indicates the absence of chemical bonds between PA66 and CNTs, demonstrating the uniform dispersion of CNTs in the PA66 matrix.

### 3.2. Thermal Properties

The addition of the CNTs into a polymer matrix has an important impact on the thermal behavior of polymer/CNTs nanocomposites, affecting the crystallization and melting temperatures and the degree of crystallinity [30] and, consequently, the processability and the mechanical properties of the end-user parts [31,32,33,34].

**Figure 3:**
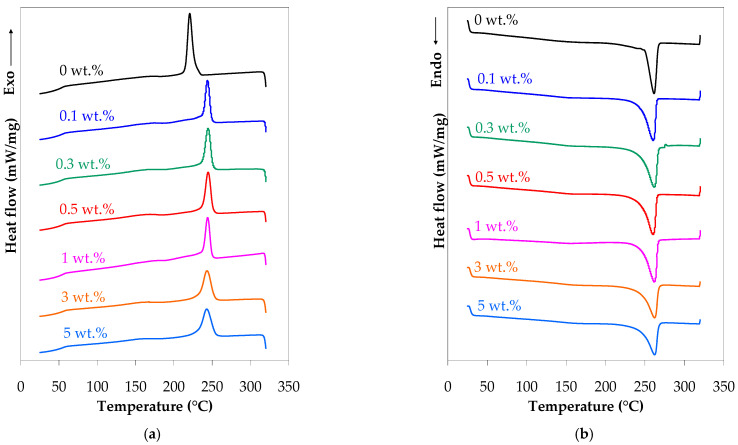

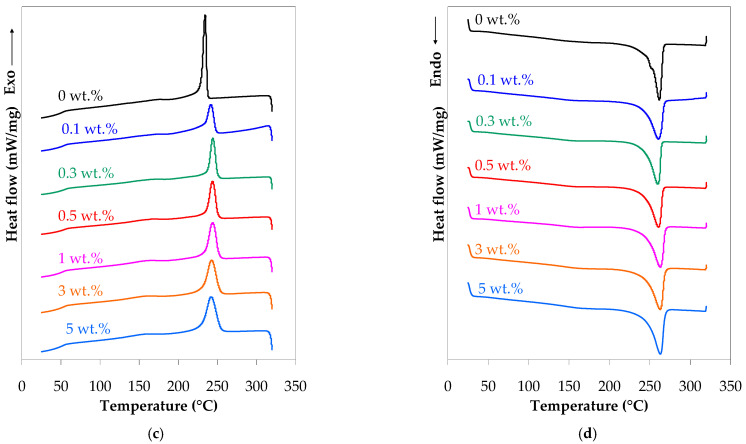
DSC thermograms of PA66 and PA66 nanocomposite: (**a**) pellets first cooling, (**b**) pellets second heating, (**c**) injection-molded samples first cooling, and (**d**) injection-molded samples second heating.

Figure 3a,b presents the DSC thermograms of the PA66 and PA66 nanocomposite pellets from the first cooling and second heating scans, while the thermal properties are summarized in Table 1.

The analysis of the cooling behavior shows a significant nucleation effect of carbon nanotubes (Figure 3a). The PA66 matrix exhibits a single crystallization peak at 222 °C. The incorporation of CNTs shifted the crystallization temperature to higher values, around 243–245 °C independent of the CNT content (Table 1), indicating that CNTs restricted the mobility of the polymer chains. Moreover, the PA66 matrix and PA66 nanocomposite pellets display a glass transition at 52–53 °C (Table 1). This effect of nanotubes on the crystallization behavior of polymer/CNT nanocomposites is well-known and has been extensively studied [14,65,91,92,93,94,95,96,97,98]. The results of the present study are consistent with the general increasing tendency in the crystallization temperature for PA66 nanocomposites reported by other authors [63,64,65,67,68,69,70], which holds true independently of the CNT content (0.05 to 10 wt.%%), the type of nanotube functionalization, or the processing methods.

Analysis of the heating curves in Figure 3b indicates that the melting behavior (e.g., melting enthalpy) and melting temperature of the PA66 nanocomposites were not affected by the addition of CNTs. The melting temperature of PA66 and its nanocomposites was in the range of 260–262 °C (Tabel 1). Additionally, the melting enthalpy increased from 86 J/g to 88–94 J/g with the inclusion of CNTs. Based on the melting enthalpy, the crystallinity of the PA66 nanocomposites varied between 46–48% and slightly increased with increasing CNT content (about 5–9% higher as compared with the neat PA66). However, no clear dependency on the CNT content could be determined. These findings are also in agreement with the results reported in the literature for PA66/CNT nanocomposites [62,63,66,67,68,69]. Studies on the effect of CNTs on the thermal behavior of PA66 reported no significant difference in the peak melting temperatures of the PA66 nanocomposites with increasing CNTs [62,63]. On the other hand, investigations on the crystallinity of the PA66 nanocomposite showed an increase (e.g., +14% at 0.3 wt.% [66]; +26% at 0.5 wt.% functionalized CNTs [62]; +21% [63], and +9% [67] at 1 wt.%, and +12% at 2 wt.% [68,69]) or no clear dependency (at 0.5 to 2 wt.% [62] or 1 to 10 wt.% [70]), as compared to the neat PA66.

The neat PA66 displays a processing window of about 39 °C, while the processing window of the PA66 nanocomposites narrows to about 14–18 °C (Table 1). This indicates that the neat PA66 is relatively easier to process than the PA66/CNT nanocomposite.

Figure 3c,d presents the DSC thermograms of the injection-molded PA66 and PA66 nanocomposite samples from the first cooling and second heating scans. The injection molding does not have a significant effect on the thermal behavior of the PA66 and its nanocomposites (melt temperature 259–262 °C and melting enthalpy 88–93 J/g). The crystallization temperature of the PA66 injection-molded samples was about 14 °C higher than that of the corresponding pellets. Although the crystallization temperature of the injection-molded nanocomposites was higher than that of the pellets (about 5–8 °C at 0.3–1 wt.%), there was no clear trend with CNTs. This could be explained by the fact that the cooling during injection molding occurs at a higher rate than during the extrusion process and CNTs restrict the mobility of polymer chains. On the other hand, it was observed that the crystallinity of the injection-molded parts (45–49%) is only slightly different from that of the pellets; this indicates that the mold temperature, which is above the glass-transition temperature, promotes an optimal level of crystallization.

Regarding thermal annealing, Table 1 indicates some small and inconsistent variations in the thermal properties of the injection-molded samples due to thermal annealing, particularly at low CNT loadings (a decrease in the crystallization temperature by 7–8 °C and an increase in the crystallinity by 3–8% at 0.3–1 wt.%). This suggests that an efficient crystallization rate was achieved during the injection molding process, and the crystals have undergone some transformation.

### 3.3. Physical Properties

Table 2 presents the solid-state density for PA66 and PA66/CNTs nanocomposite pellets and the corresponding injection-molded samples. The addition of CNTs increased the density of the nanocomposites as compared with the PA66 matrix, and the effect is statistically significant, as shown by the ANOVA. On the other hand, the injection-molded samples have a higher density as compared with the pellets, which is explained by the fact that during the injection molding process, the nanocomposites cool under high pressure, resulting in a denser structure.

For pellets, the addition of 0.1 and 5 wt.% of CNTs increases the PA66 nanocomposite density by about 4% and 7%, respectively, as compared with the neat PA66. This is likely due to the variation of the crystallinity [5,99,100] and CNT loading. The increase in density with increasing CNT loading correlates with the increase in crystallinity of the nanocomposites, as shown in Table 1. However, as indicated by the ANOVA with Tukey test in Table 2, the effect of CNTs is not significant for all loadings.

### 3.4. Rheological Properties

#### 3.4.1. Melt Flow Index

The melt flow index has practical implications, being used to investigate the material quality. In general, a high value of MFI indicates lower viscosity and better processability [101,102]. However, a material with a higher MFI than expected may experience flashing during the injection molding process, while a material with lower MFI values than expected may result in incomplete filling, leading, in both cases, to an increase in the rejection rate. The literature review indicates that adding CNTs to polymers decreases the MFI of the polymer/CNT nanocomposites [9,10,11,13,14,103].

The MFI results of the PA66 and PA66/CNTs nanocomposites are reported in Table 3. Incorporating CNTs into PA66 significantly decreases the MFI of the PA66 nanocomposites. For example, at 275 °C, the MFI of the PA66 is about 36 g/10 min and decreased to about 24, 18, and 3.5 g/10 min with the addition of 0.5, 1, and 3 wt.% CNTs, respectively. The addition of CNTs into the polymer matrix reduces the chain mobility, blocking the movement of the polymer chains during flow, and increases the flow resistance [11,13,104]. This is confirmed by the fact that for the PA66 nanocomposite with 5 wt.% CNTs, the MFI could not be measured at 1.2 kg load even when the extruded temperature was increased. Therefore, the MFI was measured at 3.8 kg load. The effect of temperature on the MFI values is more important at higher CNT loading, as shown in Table 3. This is because a higher melt temperature facilitates the movement of carbon nanotubes and polymer chains, and thus, the MFI increases [12,20,105].

#### 3.4.2. Apparent Melt Shear Viscosity

**Figure 4:**
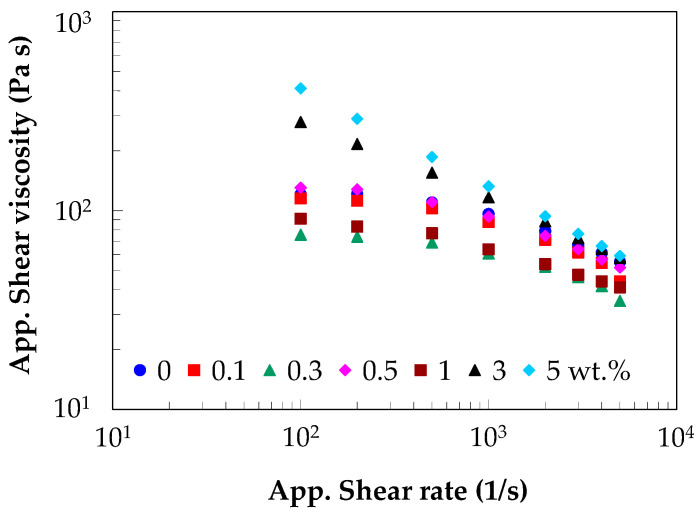
Effect of the CNT wt.% on the apparent melt shear viscosity.

The apparent flow curves (i.e., shear viscosity vs. shear rate) for the PA66 and PA66/CNT nanocomposites are shown in Figure 4. At low shear rates, the apparent shear viscosity of the PA matrix shows a Newtonian plateau followed by shear-thinning behavior at high shear rates. On the other hand, Figure 4 indicates a progressive reduction in the plateau with increasing CNT loading, and the presence of the shear-thinning behavior even at low shear rates, as is the case of the nanocomposites with 3 and 5 wt.% CNTs. As shown in Figure 4, in general, low content of CNTs (0.1 to 1 wt.%) decreases the melt shear viscosity of the PA66 matrix, while high content (3 and 5 wt.%) of CNTs increases the viscosity. At low CNT wt.%, the nanotubes are uniformly dispersed and isolated, and the motion of the polymer chains is not restricted but even increased. Therefore, the viscosity of the PA66 nanocomposites is almost independent of or decreased by the CNTs.

The viscosity of the PA66 matrix significantly increased with increasing CNT loading (e.g., 3 and 5 wt.%), displaying shear-thinning behavior even at low shear rates. For example, at a shear rate of 100 s^−1^, the PA66 shows a viscosity of about 121 Pa·s, while the PA66/CNT nanocomposite with 5 wt.% CNTs shows about 411 Pa·s, more than a threefold increase. However, at high shear rates, the difference between the PA66 matrix and the corresponding nanocomposites is less evident, as the shear-thinning behavior is dominant and the CNT-CNT interactions govern the rheological behavior of the nanocomposites.

The power-law model was used to further analyze the rheological behavior of the PA66 and PA66/CNT nanocomposites in the shear-thinning domain (e.g., 500–5000 s^−1^ [106])

(2) $$\eta_{a}=K\cdot {\dot{\gamma }}_{a}^{n-1}\;(\mathrm{Pa}\cdot s),$$

where $\eta_{a}$ is the apparent shear viscosity, ${\dot{\gamma }}_{a}$ is the apparent shear rate, *K* is the consistency coefficient, and *n* is the shear-thinning index.

The values for *K* and *n* are listed in Table 4. The relationship between the apparent shear viscosity and shear rate is very well characterized by the power-law as demonstrated by the *R*^2^ value that is close to 1. The shear-thinning index decreases with increasing CNT loading from 0 to 5 wt.%, indicating that the shear-thinning behavior becomes dominant at higher CNT loadings.

### 3.5. Microstructural Properties

**Figure 5:**
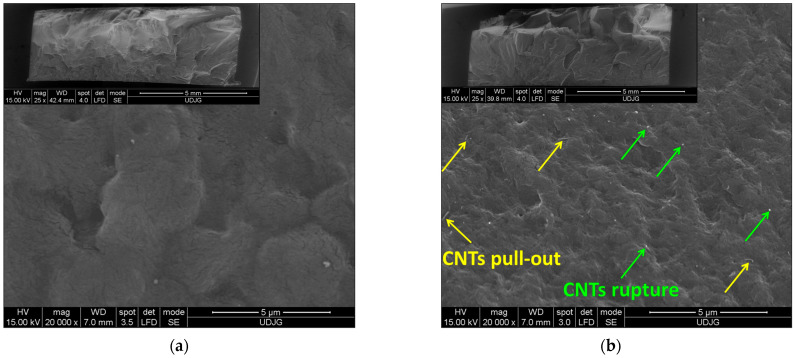

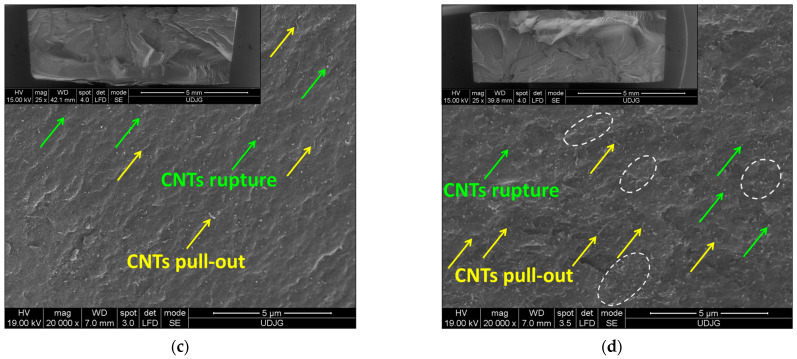
SEM images of the cryo-fractured cross-section of the PA66 (**a**) and PA66 nanocomposites with (**b**) 0.1, (**c**) 1, and (**d**) 5 wt.% CNTs.

Figure 5 shows the SEM micrographs of the cryo-fractured PA66 and PA66 nanocomposites with 0.1, 1, and 5 wt.% CNTs. It should be noted that the micrographs correspond to the core area of the injection-molded samples, perpendicular to the flow direction. At low magnification (×25), the micrographs of the PA66 and PA66 nanocomposites show a typical brittle-like fracture surface without voids. At higher magnification (×20,000), the SEM images indicate that the CNTs are well embedded and mostly uniformly dispersed within the PA66 matrix. In the case of PA66 nanocomposite with 5 wt.%, few CNT agglomerates are observed (marked as ellipses in Figure 5d). Additionally, the adhesion between the PA66 matrix and CNTs is good, as only a few nanotubes were pulled out from the PA66 matrix or exposed (marked as arrows), particularly at 5 wt.% CNTs (Figure 5). This result correlates with the FT-IR results in Figure 2.

**Figure 6:**
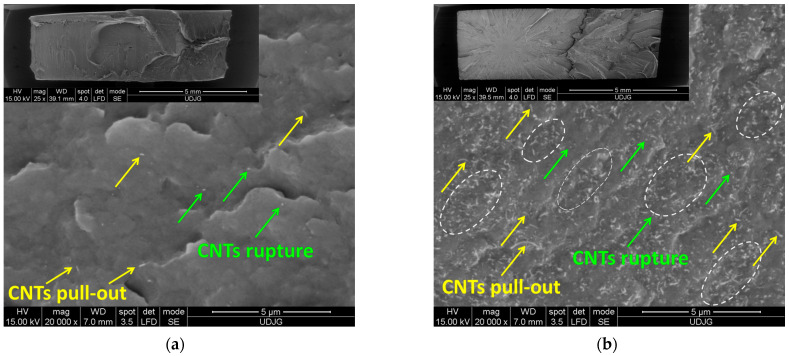
SEM images of the cross-section of the PA66 nanocomposites with (**a**) 0.1 and (**b**) 5 wt.% CNTs after tensile testing.

Figure 6 shows the SEM micrographs of the fractured PA66 nanocomposite samples with 0.1 and 5 wt.% CNTs after tensile testing. At 0.1 wt.%, the SEM images reveal the same morphological properties as those in the case of cryo-fractured surfaces (Figure 6a). However, at 5 wt.%, the SEM image shows a significant number of CNT agglomerates (marked as ellipses in Figure 6b). The main reason is that on the cryo-fractured surface (Figure 5d), only the CNTs with one end on the surface are visible. On the other hand, the increased number of pull-outs observed in Figure 6b suggests that the interfacial adhesion between the CNTs and the PA66 matrix was weakened during the tensile tests.

### 3.6. Mechanical Properties

#### 3.6.1. Tensile Properties

**Figure 7:**
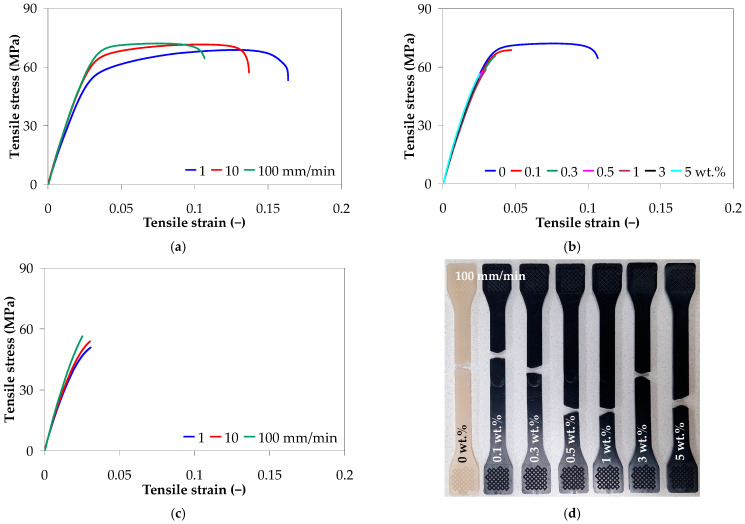
Representative tensile stress–strain curves: (**a**) effect of crosshead speed for the neat PA66, (**b**) effect of CNTs at 100 mm/min, (**c**) effect of crosshead speed for the PA66 nanocomposite with 5 wt.% CNTs, and (**d**) tested samples at 100 mm/min.

Figure 7 shows representative tensile stress–strain curves for the neat PA66 and PA66/CNT nanocomposites, tested under different conditions. The injection-molded PA66 displays moderate ductile behavior with an elongation at break of about 0.17 for a crosshead speed of 1 mm/min, which decreased to 0.11 with increasing crosshead speed to 100 mm/min (Figure 7a). However, the crosshead speed had no major influence on the tensile strength of PA66 as its value remained almost constant with increasing crosshead speed from 1 to 100 mm/min. In contrast, the PA66 nanocomposites exhibit brittle behavior, with a significant reduction in tensile strength and elongation at break, as shown in Figure 7b. Nonetheless, the linear region of the stress–strain curves is not visibly affected by the addition of CNTs.

For example, at 100 mm/min, the tensile strength and elongation at break of the PA66 nanocomposite with 5 wt.% decreased by 21% and 77%, respectively, as compared to the PA66 (Figure 7b). The effect of crosshead speed on the load-bearing capacity of PA66 nanocomposite with 5 wt.% is less significant, as illustrated in Figure 7c.

**Figure 8:**
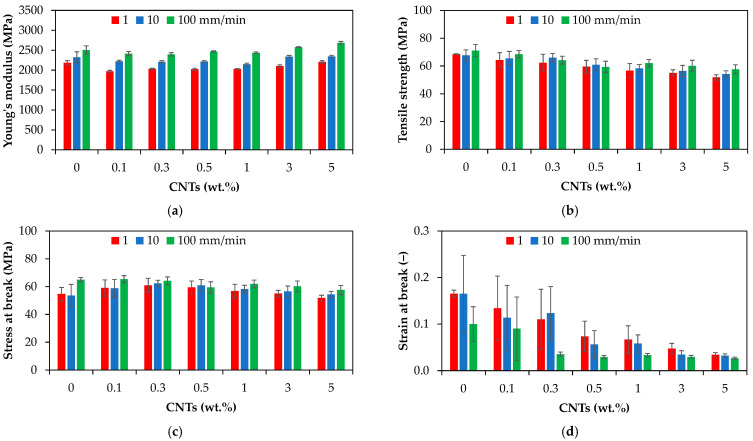
Tensile properties of PA66 and PA66 nanocomposites: (**a**) Young’s modulus, (**b**) tensile strength, (**c**) stress at break, and (**d**) strain at break.

Figure 8 presents the tensile properties of the PA66 and PA66 nanocomposites (e.g., Young’s modulus, tensile strength, stress at break, and strain at break) as a function of CNT loading and crosshead speed.

To further assess the effect of the CNTs and crosshead speed on the tensile properties, the experimental data were analyzed using the main effect plots (Figure S1 of Supplementary Materials) and ANOVA (Tables S1–S4 of Supplementary Materials). Table 5 summarizes the results from the ANOVA in terms of *F*-value and *p*-value.

As shown in Figure 8, the inclusion of carbon nanotubes (0.1 to 5 wt.%) in the PA66 matrix could not improve the tensile modulus to a great extent. The Young’s modulus of the PA66 nanocomposites was in the range of 1970 ± 30 to 2690 ± 60 MPa, depending on the CNT wt.% and crosshead speed, as shown in Figure 8a. The tensile modulus of the injection-molded PA66 nanocomposites slightly decreased at low CNT content (0.1 to 1 wt.%) as compared with the neat PA66, while an increase was measured at higher content (+3% and +7% for 3 and 5 wt.%, respectively). Tensile strengths ranging from 51.77 ± 1.98 to 68.42 ± 2.76 MPa were measured for the PA66 nanocomposites, slightly decreasing with increasing CNT loading, as shown in Figure 8b. Similar to the tensile strength, the stress at break of the PA66 nanocomposites displays the same decreasing tendency.

Both crosshead speed and CNT loading have a statistically significant effect on the mechanical properties (*p*-value < 0.05, Table 5). However, the effect and contribution depend on the type of mechanical properties (Tables S1–S3 of Supplementary Materials). The Young’s modulus, tensile strength, and stress at break of the PA66 nanocomposite display the same increasing behavior with increasing crosshead speed from 1 to 100 mm/min (Figure S1). The increase in tensile properties with increasing crosshead speed suggests that the interfacial interactions allow an effective stress transfer between PA66 and CNTs. It should be noted that, several studies have reported an increase in the Young’s modulus and tensile strength of PA66 with the addition of CNTs [8,62,63,66]. The decrease in tensile modulus and tensile strength could be explained by the presence of CNT agglomerates and the wreaked interface between the CNTs and PA66 matrix, as during the tensile loading of the nanocomposites, the interface is subjected to shear stress.

The strain at break decreased with increasing both CNT content and crosshead speed, and the effect of carbon nanotubes (76% contribution) is more significant than the effect of crosshead speed (16% contribution), as indicated by the higher *F*-value in Table 5 and Table S4, and Figure S1. At 1 mm/min, as the CNT loading increases from 0 to 5 wt.%, the elongation at break gradually decreases from about 0.165 to 0.034, as shown in Figure 8d. These results align with the general trend reported in the literature for the injection-molded polymer/CNT nanocomposites [9,12,14], and was also observed by Qiu et al. [63] for the PA66/CNT composite films with 1 wt.% CNTs. This can be explained by the fact that the high aspect ratio of CNTs hinders the segmental flexibility [41] and the ordered arrangement of the polymer chains, leading to stress concentration at the interface between CNTs and the PA66 matrix [5,107,108], and the decrease in elongation at break. Moreover, as reported in the literature for polymer nanocomposites [5,109,110,111], the connection between the polymer chains is restricted due to the increase in crystallinity with increasing CNT content, resulting in decreased ductility of the PA66. Finally, the presence of CNT agglomerates (Figure 6) can also explain the gradual decline in the elongation at break of the PA66 nanocomposites as compared with the neat PA66 [41].

**Figure 9:**
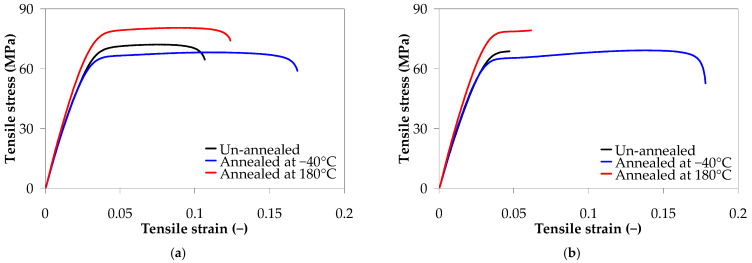

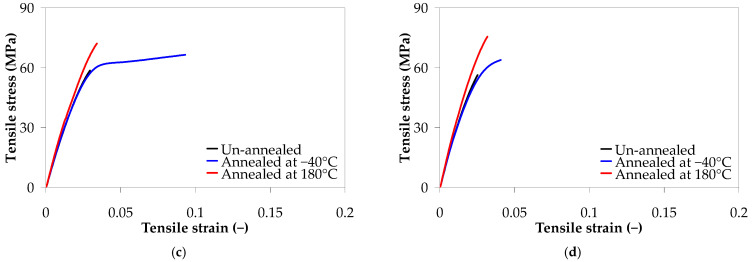
Effect of annealing on the tensile stress–strain curves of (**a**) PA66, (**b**) PA66 nanocomposite with 0.1 wt.% CNTs, (**c**) PA66 nanocomposite with 1 wt.% CNTs, and (**d**) PA66 nanocomposite with 5 wt.% CNTs.

Figure 9 shows the stress–strain curves of PA66 and PA66 nanocomposites before and after annealing. The tensile tests reveal that the ductility of the injection-molded samples is improved after annealing, especially for samples annealed at −40 °C, as shown in Figure 9. For example, after annealing at −40 °C for 1 h, the elongation at break of the annealed PA66 reached a value of 0.17, which is about 58% higher than the unannealed sample, while the elongation of PA66 nanocomposite with 5 wt.% of CNTs reached a value of 0.05, which is about 61% higher than that of the unannealed sample. On the other hand, the results show that the load-bearing capacity of the annealed nanocomposites is improved, indicating an increase in the interfacial adhesion between the CNTs and PA66 matrix.

**Figure 10:**
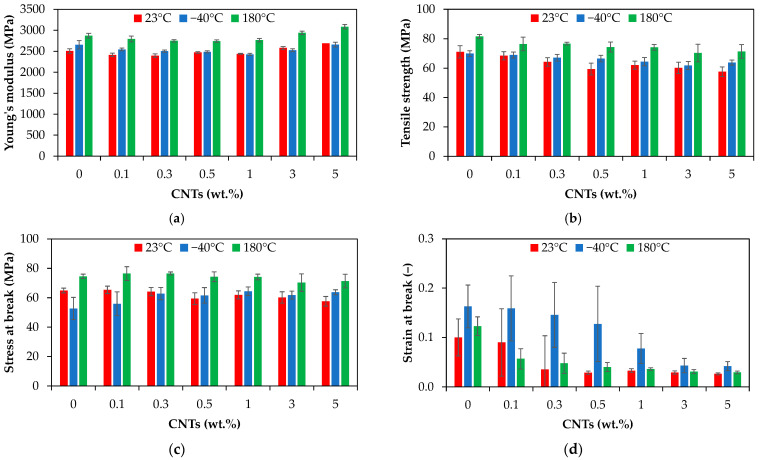
Effect of thermal annealing on the tensile properties of as-molded PA66 and PA66 nanocomposites: (**a**) Young’s modulus, (**b**) tensile strength, (**c**) stress at break, and (**d**) strain at break.

Figure 10 shows the effect of thermal annealing on the mechanical properties of PA66 and PA66 nanocomposites under tensile testing. The unannealed samples are the reference samples that can also be considered annealed at room temperature (23 °C).

As observed in Figure 10, the mechanical properties increased after annealing at 180 °C for 1 h. As the annealing temperature increased to 180 °C, the Young’s modulus, tensile strength, and stress at break of the PA66 injection-molded samples increased to 2871.98 ± 54.46 MPa, 81.57 ± 1.34 MPa, and 74.56 ± 1.50 MPa, respectively, as compared to unannealed PA66 samples (15% increase). The Young’s modulus and tensile strength of PA66 nanocomposites with 5 wt.% CNT reached 3085.02 ± 55.42 MPa (15% increase) and 71.34 ± 4.63 MPa (24% increase), respectively, as compared to reference nanocomposites. For the samples annealed at −40 °C for 1 h, the tensile modulus, tensile strength, and stress at break were almost independent of annealing. However, the samples annealed at −40 °C for 1 h, although displaying high variability in the elongation at break, exhibited higher ductility than those annealed at 180 °C or unannealed.

The increase in mechanical properties could be related to the improvement in the interfacial adhesion between the CNTs and PA66 matrix and mobility of the polymer chain, and the reduction in residual stresses. The effect of residual stresses is more important in the case of nanocomposites due to the confining effect of CNTs. In general, annealing above the glass transition can effectively reduce the residual stresses developed during the injection molding process, which mainly originate from the rapid cooling [112,113,114,115]. During the heating of nanocomposites, CNTs can rearrange and build additional networks, leading to an increase in mechanical properties, especially tensile modulus.

Table 6 summarizes the *F*-value and *p*-value for the ANOVA of the tensile properties, based on the ANOVA results (Tables S5–S8 of Supplementary Material). Table 6 and the main effect plots in Figure S2 (see Supplementary Materials) indicate that the annealing temperatures have a statistically positive effect on the mechanical properties (*p*-value = 0.00).

#### 3.6.2. Bending Properties

**Figure 11:**
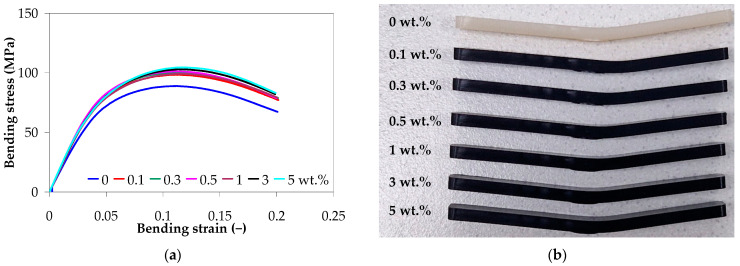
(**a**) Representative stress–strain curves for unannealed PA66 and PA66 nanocomposites under three-point bending, and (**b**) three-point bending samples at the end of the test.

Figure 11a shows the representative stress–strain curves for unannealed PA66 and PA66 nanocomposites under three-point bending. The PA66 and PA66 nanocomposites exhibit non-linear behavior with permanent plastic deformation at the end of the bending test that corresponds to a 20% strain (Figure 11b). The addition of CNTs slightly improved the bending behavior of the PA66 nanocomposites, as shown in Figure 11a. The bending strength of the PA66 nanocomposite with 0.1 wt.% and 5 wt.% is 7% (98.20 ± 1.21 MPa) and 13% (104.16 ± 1.03 MPa), respectively, higher than that of the neat PA66.

**Figure 12:**
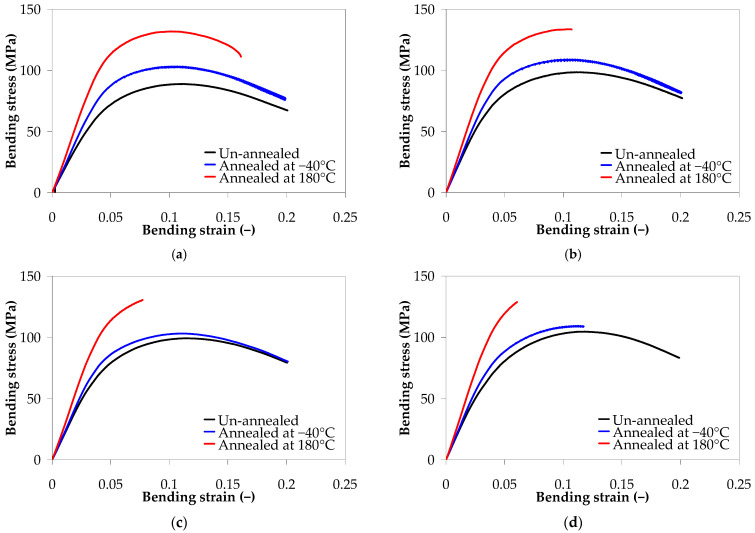
Effect of annealing on the three-point bending stress–strain curves of (**a**) neat PA66, (**b**) PA66 nanocomposite with 0.1 wt.% CNTs, (**c**) PA66 nanocomposite with 1 wt.% CNTs, and (**d**) PA66 nanocomposite with 5 wt.% CNTs.

Figure 12 presents the effect of thermal annealing on the mechanical behavior of PA66 and PA66 nanocomposites under three-point bending. In general, annealed samples display higher load-bearing capacity as compared with the unannealed samples, and the effect is more evident for the samples annealed at 180 °C. On the other hand, it is observed that the nanocomposite samples with 5 wt.% annealed at 180 °C for 1 h have a brittle behavior, exhibiting brittle fracture after reaching the maximum strength before the bending strain reached 20%.

**Figure 13:**
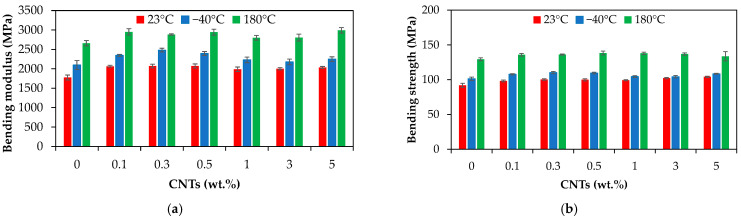
Bending mechanical properties of the annealed and unannealed PA66 and PA66 nanocomposites with different CNT loadings: (**a**) bending modulus and (**b**) bending strength.

Figure 13 compares the bending modulus and bending strength for unannealed and annealed PA66 and PA66 nanocomposite samples. In general, the annealed samples have higher mechanical properties under bending as compared with the unannealed samples, and the effect is more important for the samples annealed at 180 °C. For the neat PA66 annealed at 180 °C for 1 h, the bending modulus and strength increased by about 50% and 41%, respectively, while for the PA66 nanocomposite with 5 wt.%, they increased by about 47% and 28%, respectively, when compared to the unannealed samples.

To better understand the effect of the annealing temperature on the mechanical properties under bending, the main effect plots were constructed as shown in Figure S3 (Supplementary Materials), and the data were analyzed using ANOVA, as reported in Tables S9 and S10 (Supplementary Materials).

The results of the ANOVA are summarized in Table 7. The effect of temperature on the bending modulus and bending strength is statistically significant (*p*-value = 0.00, Table 7); all annealed samples present increased modulus and strength as compared to the unannealed ones. On the other hand, although CNT loading has a significant effect on the bending properties (*p*-value = 0.00 for bending modulus, *p*-value = 0.01 for bending strength), the contribution is marginal (e.g., about 7% and 3% on the bending modulus and bending strength, respectively) as compared with the annealing temperature (e.g., about 91% and 96% on the bending modulus and bending strength, respectively).

## 4. Conclusions

This study reports on the effect of CNTs on the physical, thermal, rheological, morphological, and mechanical properties of the PA66 nanocomposites. First, the physical, thermal, and rheological properties of PA66 and PA66 nanocomposites with 0.1–5 wt.% CNTs were assessed on pellets. Then, PA66 and PA66 nanocomposite samples were prepared by injection molding and tested in tensile and three-point bending modes, and the morphological properties were assessed before and after mechanical testing. In addition, the effect of short-thermal annealing on the thermal and mechanical properties of PA66 nanocomposites was assessed. Based on the experimental results, the following conclusions have been drawn:

- The analysis of the DSC thermal behavior showed a significant nucleation effect of CNTs and no significant change or increase in the glass transition, melt temperature, and crystallinity of PA66 nanocomposites. Incorporating CNTs into PA66 led to a shift in the crystallization temperature to higher values (by approximately 20–25 °C and 7–17 °C for the pellets and injection-molded samples, respectively), making the PA66 nanocomposites more thermally stable than the neat PA66.
- The FT-IR analysis showed that there are no significant variations in either the peak positions or relative intensities for the neat PA66 and PA66 nanocomposites, indicating the absence of chemical bonds between PA66 and CNTs and, consequently, the uniform dispersion of CNTs in the PA66 matrix.
- SEM micrographs reported a good distribution of CNTs in the PA66 matrix, even though some agglomerates were identified with increasing CNT content.
- The rheological behavior of the PA66 composites investigated using MFI and capillary rheology indicates that the addition of CNTs decreased the MFI and increased in the melt shear viscosity, particularly at CNT contents higher than 1 wt.%. The PA66 nanocomposites exhibit shear-thinning behavior, and this effect is more pronounced at higher CNT contents (e.g., shear-tinning index decreased from 0.7 to 0.5 with increasing CNTs from 0.1 to 5 wt.%), indicating that the PA66 nanocomposites can be easily processed at higher shear rates.
- The inclusion of CNTs (0.1 to 5 wt.%) in PA66 matrix did not improve the tensile modulus to a great extent, while it decreased the ultimate tensile strength, stress, and elongation at break of PA66 nanocomposites under tension. On the other hand, CNTs positively influenced the mechanical behavior under bending (+15% increase at 5 wt.%). Among the nanocomposites, PA66/CNT with 5 wt.% exhibited the optimal mechanical performance in terms of tensile strength (58 MPa), tensile modulus (2689 MPa), bending modulus (2072 MPa), and bending strength (104 MPa).
- The experimental results showcase improvements in mechanical properties, including the elongation at break of the injection-molded PA66 nanocomposites under both tensile and bending conditions after thermal annealing at −40 °C or 180 °C for only one hour.

In conclusion, the experimental results reveal the complex relationship between CNTs and the enhancement of mechanical properties, highlighting the potential of thermal annealing in improving the mechanical performance of PA66 nanocomposites, which will be further investigated. As the present findings contribute to the understanding of the structure–property–processability of PA66/CNT nanocomposites, future studies will also explore the electrical behavior of PA66 nanocomposites and identify industrial applications, such as injection molding of lightweight automotive or aerospace components that require high strength at elevated temperatures.

## Figures and Tables

**Table 1:** The thermal properties of PA66 and PA66 nanocomposite.

PA66/CNT (wt.%)			0	0.1	0.3	0.5	1	3	5
First Cooling	*T_g_* (°C)	P	52.6	51.8	52.8	52.8	52.2	53.3	53.0
		IM	52.0	50.9	53.2	51.4	50.5	52.3	52.3
		Ann. (−40 °C)	52.1	52.7	53.5	52.6	52.7	54.3	53.5
		Ann. (180 °C)	52.3	51.3	52.8	53.2	53.3	53.4	51.4
	*T_c_* (°C)	P	221.6	244.5	245.2	245.4	244.7	243.7	243.4
		IM	235.7	242.4	250.6	251.0	252.6	243.3	242.8
		Ann. (−40 °C)	236.0	243.5	243.9	245.3	244.0	244.2	242.7
		Ann. (180 °C)	235.3	243.7	243.3	244.5	244.3	243.0	242.7
Second Heating	*T_m_* (°C)	P	260.7	259.5	260.9	259.5	261.1	261.6	261.7
		IM	260.7	259.9	259.1	259.9	262.1	261.7	262.1
		Ann. (−40 °C)	261.1	260.5	260.8	260.8	261.4	261.2	262.3
		Ann. (180 °C)	261.5	262.0	260.8	262.0	260.7	262.5	262.8
	Δ*H_m_ *(J/g)	P	86	92	91	94	90	91	88
		IM	92	90	88	88	92	93	88
		Ann. (−40 °C)	90	89	91	95	96	93	92
		Ann. (180 °C)	88	91	93	94	97	92	90
Δ*T* = *T_m_* − *T_c_* (°C)		P	39.2	15.0	15.7	14.2	16.4	17.9	18.3

P—pellets; IM—injection-molded samples; Ann. (−40 °C)—annealed at −40 °C for 1 h; Ann. (180 °C)—annealed at 180 °C for 1 h.

**Table 2:** Solid density of PA66 and PA66 nanocomposite.

Samples From	Density (g/cm^3^)	CNTs (wt.%)							*p*-Value
		0	0.1	0.3	0.5	1	3	5	
Pellets	Mean *	1.0595 ^c^	1.1006 ^b^	1.1038 ^b^	1.1055 ^b^	1.1072 ^b^	1.1156 ^a,b^	1.1333 ^a^	0.00
	SD (−)	0.0356	0.0069	0.0082	0.0064	0.0049	0.0069	0.0056	
Injection-molded	Mean *	1.1159 ^f^	1.1183 ^e^	1.1201 ^d^	1.1209 ^d^	1.1258 ^c^	1.1362 ^b^	1.1478 ^a^	0.00
	SD (−)	0.0010	0.0003	0.0005	0.0011	0.0009	0.0015	0.0010	

* In rows, different letters show significant differences (*p*-values < 0.05) determined by Tuckey test; *p*-value determined by one-way ANOVA.

**Table 3:** Effect of the CNT wt.% on the PA66-based nanocomposites MFI.

PA66/CNTs (wt.%)	Mass Load (kg)	Extrude Temperatures (°C)	
		275	280
0	1.2	36.13 ± 0.59	35.64 ± 2.13
0.1	1.2	34.41 ± 1.04	34.37 ± 2.05
0.3	1.2	33.28 ± 0.84	32.51 ± 1.55
0.5	1.2	24.26 ± 0.62	29.21 ± 1.23
1	1.2	17.93 ± 0.49	21.79 ± 0.74
3	1.2	3.51 ± 0.15	4.42 ± 0.22
5	1.2	No flow	No flow
5	3.8	8.04 ± 0.42	8.33 ± 0.36

**Table 4:** The power-law model for the PA66 and PA66 nanocomposites.

PA/66 CNTs (wt.%)	*K* (Pa s^n^)	*n* (−)	*R* ^2^
0	743.70	0.70	0.977
0.1	686.75	0.70	0.990
0.3	311.60	0.76	0.985
0.5	895.36	0.67	0.990
1	415.15	0.73	0.999
3	2213.60	0.57	0.990
5	4086.02	0.50	1.000

**Table 5:** ANOVA results for tensile properties of PA66 and PA66 nanocomposites.

Factor	Young’s Modulus		Tensile Strength		Stress at Break		Strain at Break	
	*F*-Value	*p*-Value	*F*-Value	*p*-Value	*F*-Value	*p*-Value	*F*-Value	*p*-Value
Crosshead speed (mm/min)	189.48	0.00	10.24	0.00	11.38	0.00	12.18	0.00
CNTs (wt.%)	13.03	0.00	38.31	0.00	4.56	0.01	19.09	0.00

**Table 6:** ANOVA results for tensile properties of PA66 and PA66 nanocomposites—effect of thermal annealing.

Factor	Young’s Modulus		Tensile Strength		Stress at Break		Strain at Break	
	*F*-Value	*p*-Value	*F*-Value	*p*-Value	*F*-Value	*p*-Value	*F*-Value	*p*-Value
Annealing temperature (°C)	96.07	0.00	94.21	0.00	28.12	0.00	14.36	0.00
CNTs (wt.%)	10.92	0.00	14.98	0.00	0.49	0.81	7.01	0.00

**Table 7:** ANOVA results for bending properties of PA66 and PA66 nanocomposites—effect of thermal annealing.

Factor	Bending Modulus			Bending Strength		
	*F*-Value	*p*-Value	C (%)	*F*-Value	*p*-Value	C (%)
Annealing temperature (°C)	413.46	0.00	91.45	501.37	0.00	96.00
CNTs (wt.%)	10.89	0.00	7.22	4.96	0.01	2.85

## Data Availability

The original contributions presented in this study are included in the article/Supplementary Materials. Further inquiries can be directed to the corresponding author.

## Supplementary Materials

The following supporting information can be downloaded at: https://www.mdpi.com/article/10.3390/polym17101319/s1, Figure S1. Main effect plots for the tensile properties for PA66 and PA66 nanocomposites: (a) Young’s modulus, (b) tensile strength, (c) stress at break, and (d) strain at break, Figure S2. Main effect plots for the annealed PA66 and PA66 nanocomposites tensile properties: (a) Young’s modulus, (b) tensile strength, (c) stress at break, and (d) strain at break, Figure S3. Main effect plots for the annealed PA66 and PA66 nanocomposites tensile properties: (a) bending modulus and (b) bending strength; Table S1. ANOVA and Tukey pairwise comparisons for Young’s modulus, Table S2. ANOVA and Tukey pairwise comparisons for tensile strength, Table S3. ANOVA and Tukey pairwise comparisons for stress at break, Table S4. ANOVA and Tukey pairwise comparisons for strain at break, Table S5. ANOVA and Tukey pairwise comparisons for Young’s modulus, Table S6. ANOVA and Tukey pairwise comparisons for tensile strength, Table S7. ANOVA and Tukey pairwise comparisons for stress at break, Table S8. ANOVA and Tukey pairwise comparisons for strain at break, Table S9. ANOVA and Tukey pairwise comparisons for bending modulus, Table S10. ANOVA and Tukey pairwise comparisons for bending strength.
