# Supplementary material for: The Effect of Multi-Walled Carbon Nanotubes on the Material Properties of Polyamide 66 Nanocomposites
Source: Polymers (Basel). 2025 May 12;17(10):1319. doi: 10.3390/polym17101319 (PMC12115250; doi:10.3390/polym17101319)
Supplement: Supplementary file 1 [file polymers-17-01319-s001.zip › polymers-3609613-supplementary.pdf]

polymers

MDPI

Supplementary materials

The Effect of Multi-Walled Carbon Nanotubes on the Material Properties of Polyamide 66 Nanocomposites

Ionut-Laurentiu Sandu [1], Felicia Stan [1], Catalin Fetecau [1,*], Adriana-Madalina Turcanu [1], Alina Cantaragiu Ceoromila [2], Andrei-Mihai Prada [3] and Florin-Sandu Blaga [3]

1 Center of Excellence Polymer Processing, Dunarea de Jos University of Galati, 47 Domneasca, 800 008 Galati, Romania; laurentiu.sandu@ugal.ro (I.-L.S.), felicia.stan@ugal.ro (F.S.), madalina.constantinescu@ugal.ro (A.-M.T.)
2 Cross-Border Faculty, Dunarea de Jos University of Galati, 47 Domneasca, 800 008 Galati, Romania; alina.cantaragiu@ugal.ro (A.C.C.)
3 Industrial Engineering Department, Faculty of Managerial and Technological Engineering, University of Oradea, 1 Universitatii, 410 087, Oradea, Romania; andrei.prada@plastor.ro (A.-M.P), fblaga@uoradea.ro (F.-S.B.)
* Correspondence: catalin.fetecau@ugal.ro (C.F.)

1. Figures

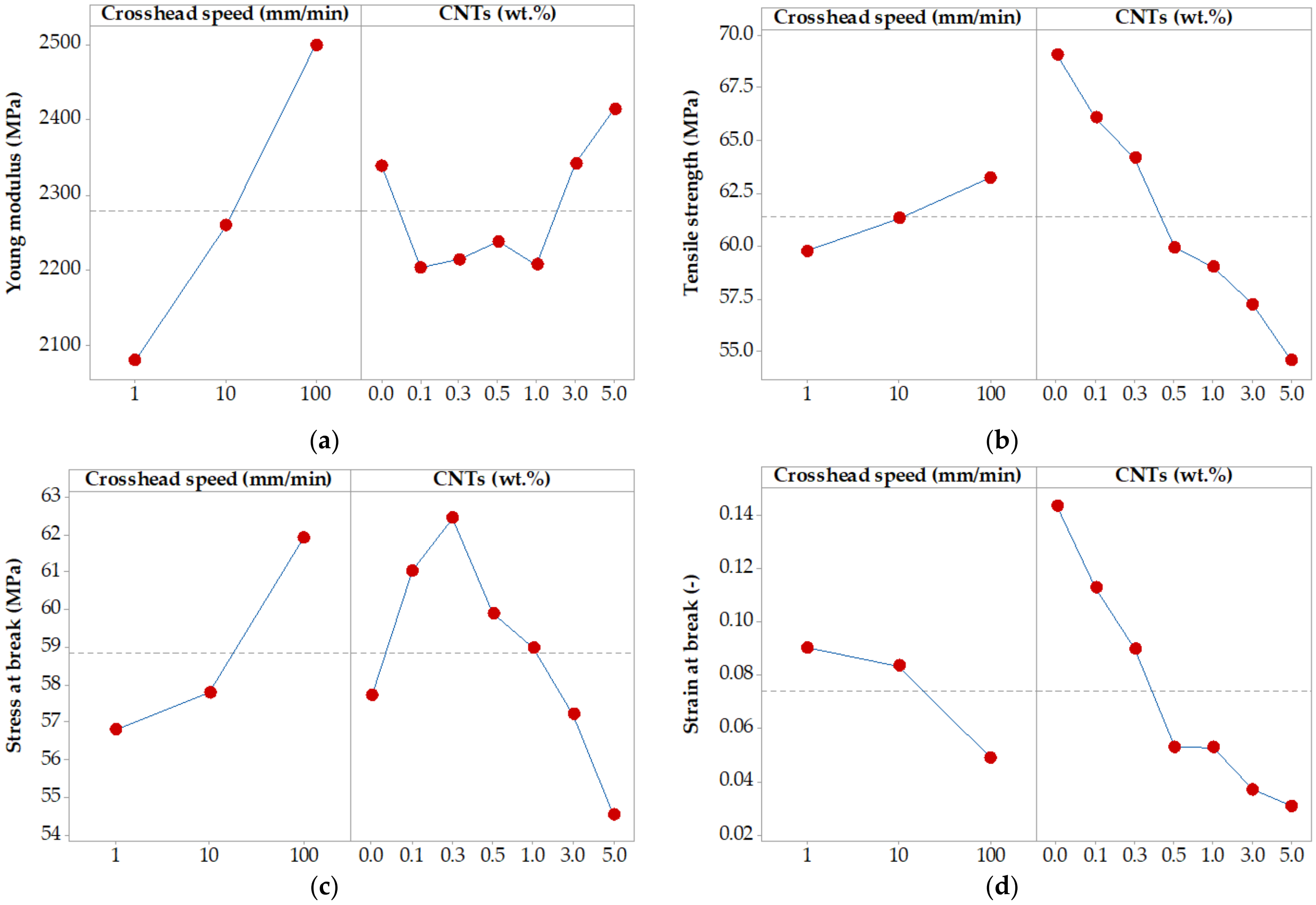

Figure S1. Main effect plots for the tensile properties for PA66 and PA66 nanocomposites: (**a**) Young's modulus, (**b**) tensile strength, (**c**) stress at break, and (**d**) strain at break.

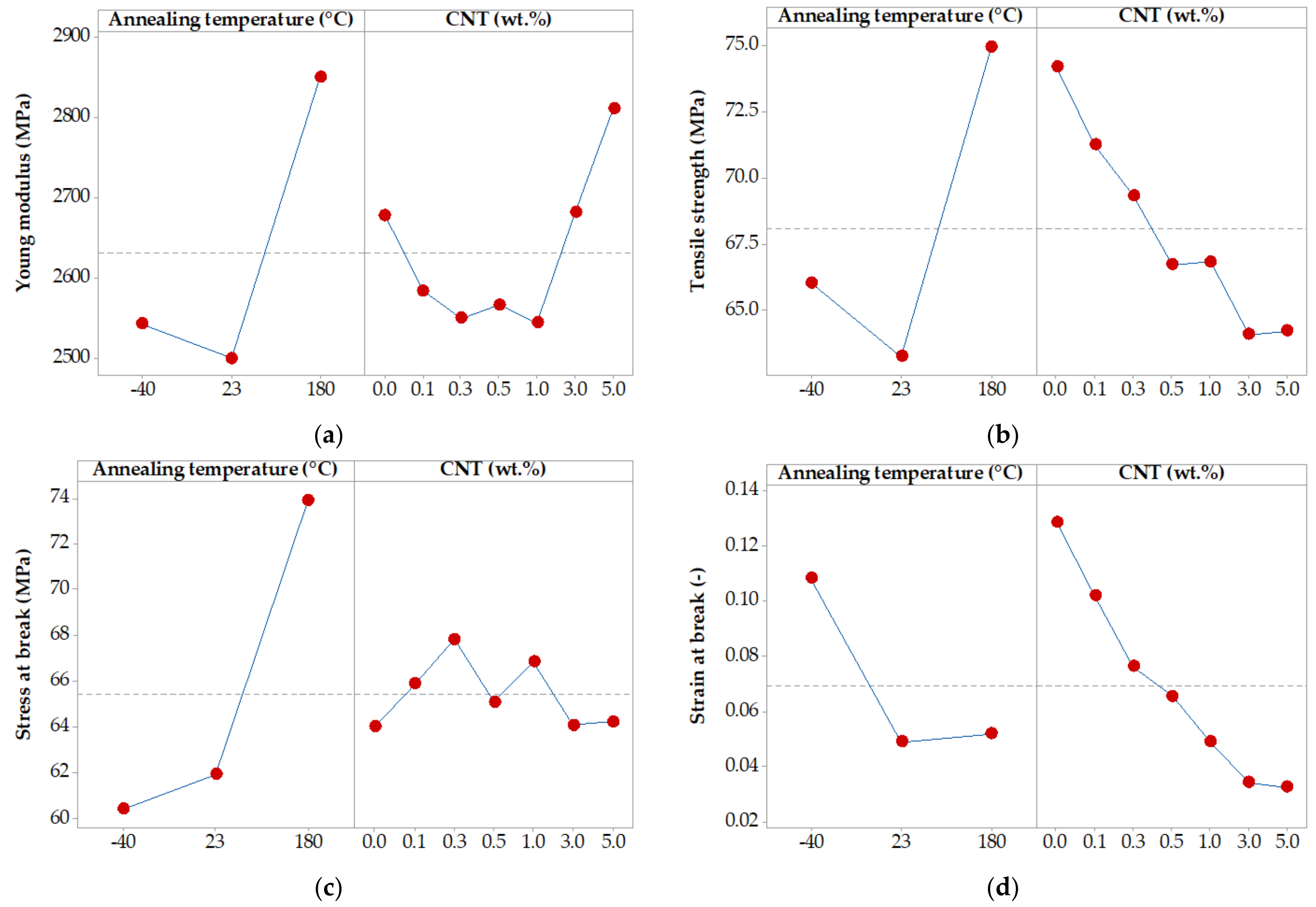

Figure S2. Main effect plots for the annealed PA66 and PA66 nanocomposites tensile properties: (**a**) Young's modulus, (**b**) tensile strength, (**c**) stress at break, and (**d**) strain at break.

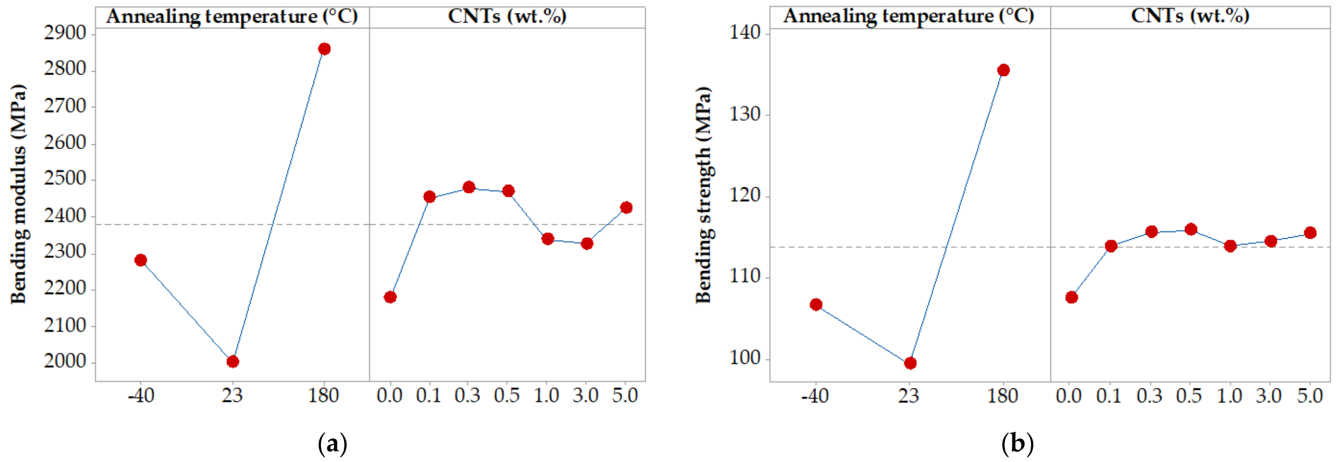

Figure S3. Main effect plots for the annealed PA66 and PA66 nanocomposites tensile properties: (**a**) bending modulus, and (**b**) bending strength.

2. Tables

2.1. Tensile properties – Effect of CNT wt.% and crosshead speed

Table S1. ANOVA and Tukey pairwise comparisons for Young's modulus.

ANOVA	DF	Adj SS	Adj MS	F-Value	*P*-Value	C (%)
Crosshead speed (mm/min)	2	621924	310962	189.48	0.00	80.78
CNTs (wt.%)	6	128313	21386	13.03	0.00	16.67
Error	12	19693	1641			2.56
Total	20	769931				100

S = 40.51; R^2 = 97.44%; R^2(adj) = 95.74%.

Tukey pairwise comparisons		Mean	Grouping*		
Crosshead speed (mm/min)	100	2499.39	A		
	10	2258.39		B	
	1	2079.38			C
CNTs (wt.%)	5	2413.83	A		
	3	2341.67	A	B	
	0	2338.20	A	B	
	0.5	2237.35		B	C
	0.3	2213.46			C
	1	2206.33			C
	0.1	2202.53			C

* Means that do not share a letter are significantly different.

Table S2. ANOVA and Tukey pairwise comparisons for tensile strength.

ANOVA	DF	Adj SS	Adj MS	F-Value	*P*-Value	C (%)
Crosshead speed (mm/min)	2	43.141	21.571	10.24	0.00	7.81
CNTs (wt.%)	6	484.26	80.709	38.31	0.00	87.62
Error	12	25.28	2.107			4.57
Total	20	552.68				100

S = 1.45; R^2 = 95.43%; R^2(adj) = 92.38%.

Tukey pairwise comparisons		Mean	Grouping*			
Crosshead speed (mm/min)	100	63.25	A			
	10	61.30	A	B		
	1	59.75		B		
CNTs (wt.%)	0	69.09	A			
	0.1	66.09	A	B		
	0.3	64.18		B		
	0.5	59.92			C	
	1	58.99			C	
	3	57.22			C	D
	5	54.54				D

* Means that do not share a letter are significantly different.

Table S3. ANOVA and Tukey pairwise comparisons for stress at break.

ANOVA	DF	Adj SS	Adj MS	F-Value	*P*-Value	C (%)
Crosshead speed (mm/min)	2	103.28	51.638	11.38	0.00	36.64
CNTs (wt.%)	6	124.13	20.688	4.56	0.01	44.04
Error	12	54.432	4.536			19.31
Total	20	281.84				100

S = 2.13; R^2 = 80.69%; R^2(adj) = 67.81%.

Tukey pairwise comparisons		Mean	Grouping*	
Crosshead speed (mm/min)	100	61.92	A	
	10	57.79		B
	1	56.80		B
CNTs (wt.%)	0.3	62.45	A	
	0.1	61.04	A	
	0.5	59.91	A	B
	1	58.99	A	B
	0	57.72	A	B
	3	57.21	A	B
	5	54.54		B

* Means that do not share a letter are significantly different.

Table S4. ANOVA and Tukey pairwise comparisons for strain at break.

ANOVA	DF	Adj SS	Adj MS	F-Value	*P*-Value	C (%)
Crosshead speed (mm/min)	2	0.01	0.00	12.18	0.00	16.14
CNTs (wt.%)	6	0.03	0.01	19.09	0.00	75.91
Error	12	0.00	0.00			7.95
Total	20	0.04				100

S = 0.017; R^2 = 92.05%; R^2(adj) = 86.75%.

Tukey pairwise comparisons		Mean	Grouping*			
Crosshead speed (mm/min)	1	0.09	A			
	10	0.08	A			
	100	0.05		B		
CNTs (wt.%)	0	0.14	A			
	0.1	0.11	A	B		
	0.3	0.09		B	C	
	0.5	0.05			C	D
	1	0.05			C	D
	3	0.04				D
	5	0.03				D

* Means that do not share a letter are significantly different.

2.2. Tensile properties – Effect of annealing and CNT wt.% at 100 mm/min

Table S5. ANOVA and Tukey pairwise comparisons for Young's modulus

ANOVA	DF	Adj SS	Adj MS	F-Value	*P*-Value	C (%)
Thermal annealing (°C)	2	509671	254836	96.07	0.00	71.26
CNTs (wt.%)	6	173770	28962	10.92	0.00	24.29
Error	12	31831	2653			4.45
Total	20	715272				100

S = 51.50; R^2 = 95.55%; R^2(adj) = 92.58%.

Tukey pairwise comparisons		Mean	Grouping*	
Thermal annealing (°C)	180	2849.16	A	
	-40	2542.13		B
	23	2499.39		B
CNTs (wt.%)	5	2811.19	A	
	3	2680.81	A	B
	0	2677.37	A	B
	0.1	2583.41		B
	0.5	2566.17		B
	0.3	2549.08		B
	1	2543.53		B

* Means that do not share a letter are significantly different.

Table S6. ANOVA and Tukey pairwise comparisons for tensile strength.

ANOVA	DF	Adj SS	Adj MS	F-Value	*P*-Value	C (%)
Thermal annealing (°C)	2	523.64	261.82	94.21	0.00	64.90
CNTs (wt.%)	6	249.84	41.64	14.98	0.00	30.97
Error	12	33.35	2.78			4.13
Total	20	806.83				100

S = 1.67; R^2 = 95.87%; R^2(adj) = 93.11%.

Tukey pairwise comparisons		Mean	Grouping*		
Thermal annealing (°C)	180	74.96	A		
	-40	66.04		B	
	23	63.25			C
CNTs (wt.%)	0	74.18	A		
	0.1	71.25	A	B	
	0.3	69.33		B	
	1	66.83		B	C
	0.5	66.71		B	C
	5	64.22			C
	3	64.07			C

* Means that do not share a letter are significantly different.

Table S7. ANOVA and Tukey pairwise comparisons for stress at break.

ANOVA	DF	Adj SS	Adj MS	F-Value	*P*-Value	C (%)
Thermal annealing (°C)	2	769.00	384.50	28.12	0.00	79.03
CNTs (wt.%)	6	39.90	6.65	0.49	0.81	4.10
Error	12	164.11	13.68			16.87
Total	20	973.02				100

S = 3.70; R^2 = 83.13%; R^2(adj) = 71.89%.

Tukey pairwise comparisons		Mean	Grouping*	
Thermal annealing (°C)	180	73.93	A	
	23	61.92		B
	-40	60.40		B
CNTs (wt.%)	0.3	67.83	A	
	1	66.83	A	
	0.1	65.88	A	
	0.5	65.08	A	
	5	64.22	A	
	3	64.07	A	
	0	64.03	A	

* Means that do not share a letter are significantly different.

Table S8. ANOVA and Tukey pairwise comparisons for strain at break.

ANOVA	DF	Adj SS	Adj MS	F-Value	*P*-Value	C (%)
Thermal annealing (°C)	2	0.02	0.01	14.36	0.00	34.70
CNTs (wt.%)	6	0.02	0.00	7.01	0.00	50.80
Error	12	0.01	0.00			14.50
Total	20	0.04				100

S = 0.02; R^2 = 85.50%; R^2(adj) = 75.83%.

Tukey pairwise comparisons		Mean	Grouping*		
Thermal annealing (°C)	-40	0.11	A		
	180	0.05		B	
	23	0.05		B	
CNTs (wt.%)	0	0.13	A		
	0.1	0.10	A	B	
	0.3	0.08	A	B	C
	0.5	0.07	A	B	C
	1	0.05		B	C
	3	0.03			C
	5	0.03			C

* Means that do not share a letter are significantly different.

2.3. 3-point bending properties – Effect of thermal annealing and CNT wt.%

Table S9. ANOVA and Tukey pairwise comparisons for bending modulus.

ANOVA	DF	Adj SS	Adj MS	F-Value	*P*-Value	C (%)
Thermal annealing (°C)	2	2690912	1345456	413.46	0.00	91.45
CNTs (wt.%)	6	212532	35422	10.89	0.00	7.22
Error	12	39049	3254			1.33
Total	20	2942493				100

S = 57.05; R^2 = 98.67%; R^2(adj) = 97.79%.

Tukey pairwise comparisons		Mean	Grouping*		
Thermal annealing (°C)	180	2860.87	A		
	-40	2281.81		B	
	23	2001.13			C
CNTs (wt.%)	0.3	2478.63	A		
	0.5	2470.16	A		
	0.1	2453.59	A		
	5	2423.82	A		
	1	2338.42	A	B	
	3	2327.06	A	B	
	0	2177.19		B	

* Means that do not share a letter are significantly different.

Table S10. ANOVA and Tukey pairwise comparisons for bending strength.

ANOVA	DF	Adj SS	Adj MS	F-Value	*P*-Value	C (%)
Thermal annealing (°C)	2	5095.58	2547.79	501.37	0.00	96.00
CNTs (wt.%)	6	151.31	25.22	4.96	0.01	2.85
Error	12	60.98	5.08			1.15
Total	20	5307.87				100

S = 2.25; R^2 = 98.85%; R^2(adj) = 98.09%.

Tukey pairwise comparisons		Mean	Grouping*		
Thermal annealing (°C)	180	135.48	A		
	-40	106.62		B	
	23	99.43			C
CNTs (wt.%)	0.5	115.94	A		
	0.3	115.59	A		
	5	115.45	A		
	3	114.53	A		
	1	113.93	A	B	
	0.1	113.92	A	B	
	0	107.53		B	

* Means that do not share a letter are significantly different.
